# Transient perivascular inflammation of the carotid artery syndrome: diagnostic value of unenhanced MRI and ultrasonography

**DOI:** 10.1093/bjrcr/uaag002

**Published:** 2026-01-25

**Authors:** Yoshisuke Kadoya, Koji Nobata, Yuzo Shimode, Daisuke Kita, Seiko Miura, Yuka Nishino, Satoshi Shibata, Tamaki Kondo, Kiyotaka Ohta, Takafumi Mochizuki, Naoto Watanabe, Koshi Shimanaka, Tetsuya Minami

**Affiliations:** Department of Radiology, Kanazawa Medical University, Ishikawa, 920-0293, Japan; Department of Radiology, Kanazawa Medical University, Ishikawa, 920-0293, Japan; Department of Otolaryngology, Anamizu General Hospital, Ishikawa, 927-0027, Japan; Department of Neurosurgery, Noto General Hospital, Ishikawa, 926-0816, Japan; Department of Radiology, Kanazawa Medical University, Ishikawa, 920-0293, Japan; Department of Radiology, Kanazawa Medical University, Ishikawa, 920-0293, Japan; Department of Radiology, Kanazawa Medical University, Ishikawa, 920-0293, Japan; Department of Radiology, Kanazawa Medical University, Ishikawa, 920-0293, Japan; Department of Radiology, Kanazawa Medical University, Ishikawa, 920-0293, Japan; Department of Radiology, Kanazawa Medical University, Ishikawa, 920-0293, Japan; Department of Radiology, Kanazawa Medical University, Ishikawa, 920-0293, Japan; Department of Internal Medicine, Anamizu General Hospital, Ishikawa, 927-0027, Japan; Department of Radiology, Kanazawa Medical University, Ishikawa, 920-0293, Japan

**Keywords:** TIPIC syndrome, carotidynia, carotid artery inflammation, magnetic resonance imaging (MRI), ultrasonography

## Abstract

Transient perivascular inflammation of the carotid artery (TIPIC) syndrome, formerly known as carotidynia, is a rare and benign condition characterized by acute unilateral neck pain and perivascular inflammation in the absence of arterial dissection or stenosis. We describe a woman in her 70s who presented with a 4-day history of right-sided neck and pharyngeal pain. Ultrasonography of the neck revealed eccentric wall thickening of the right common carotid artery with surrounding hypoechoic soft tissue, but without luminal narrowing. Because of her history of asthma, unenhanced magnetic resonance imaging was performed instead of contrast-enhanced computed tomography. Magnetic resonance angiography showed no stenosis or dissection. Fat-suppressed T2-weighted imaging demonstrated high signal intensity around the right common carotid artery, consistent with perivascular oedema, which was not present on imaging performed 7 months earlier; diffusion-weighted imaging revealed no restricted diffusion, supporting an inflammatory rather than ischaemic process. TIPIC syndrome was diagnosed based on her clinical symptoms and imaging findings. She was managed conservatively, and her symptoms resolved spontaneously within 1 week. Follow-up ultrasonography demonstrated complete resolution of the vascular wall thickening and perivascular changes. This case highlights the characteristic imaging features of TIPIC syndrome and emphasizes the importance of considering this entity in patients with unilateral neck pain.

## Introduction

Transient perivascular inflammation of the carotid artery (TIPIC) syndrome, formerly known as carotidynia, is a rare and benign condition characterized by unilateral neck pain and imaging findings of perivascular inflammation without vascular stenosis or dissection. Previously, it had been classified as a type of atypical facial pain. We report a patient with TIPIC syndrome who exhibited characteristic findings on magnetic resonance imaging (MRI) and ultrasonography (US) and was managed without using an invasive procedure.

## Case presentation

**Figure 1.**
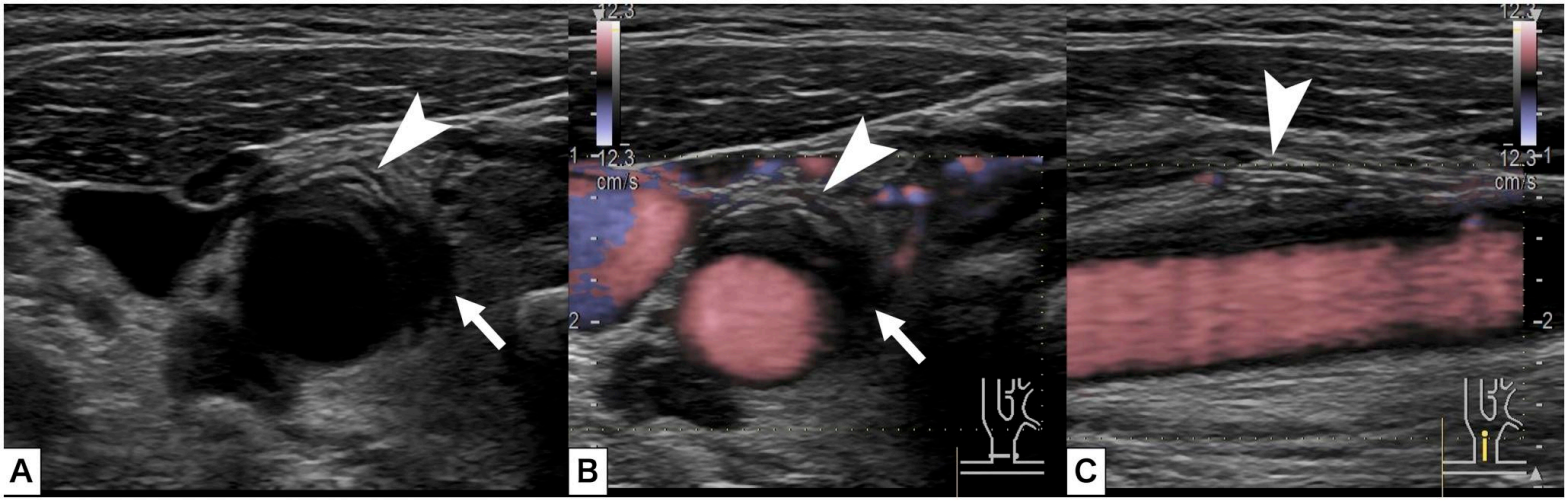
Axial ultrasonography imaging of the right common carotid artery in B-mode (A) and colour Doppler mode (B). (C) Sagittal imaging of the right common carotid artery. There is thickening of the extravascular membrane (white arrowheads) and hypoechoic areas around the vessels (white arrows). No vascular stenosis or dissection was demonstrated.

**Figure 2.**
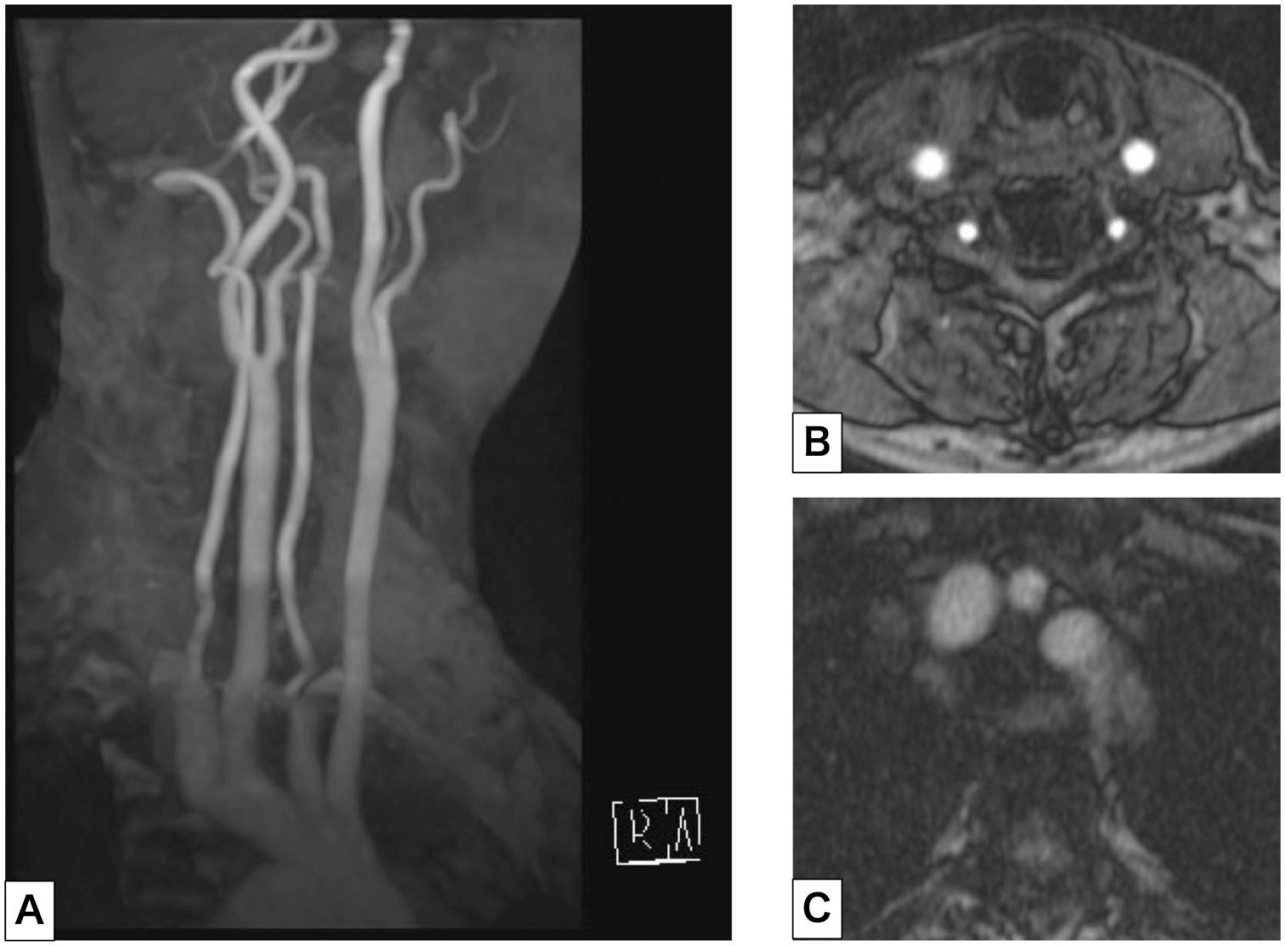
(A) Maximum intensity projection image of magnetic resonance angiography showed no arterial stenosis or dissection. (B) Axial imaging of the neck did not demonstrate dissection of the right common carotid artery. (C) At the level of the aortic arch, axial imaging showed no wall thickening or high signal intensity surrounding the origins of the aortic arch branches.

**Figure 3.**
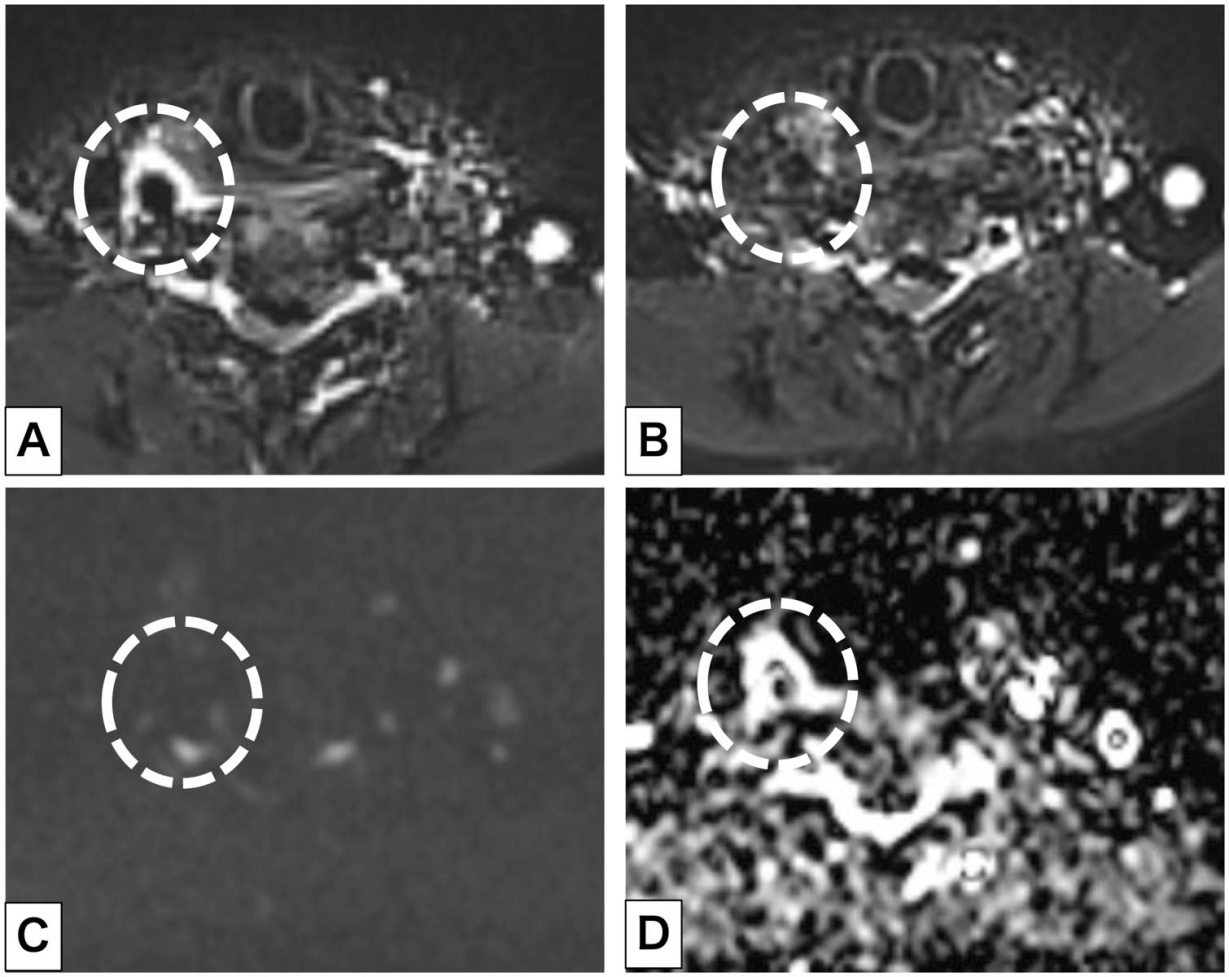
(A) Axial short tau inversion recovery imaging of the neck showed an area of high signal intensity around the right common carotid artery (CCA); the left CCA appeared normal. (B) Imaging 7 months previously showed no abnormality. (C) Diffusion-weighted imaging (*b* = 800) demonstrated no signal change around the right CCA. (D) On the apparent diffusion coefficient map, a high-signal area was seen around the right CCA. The dashed circle indicates the right CCA.

A woman in her 70s presented with a 4-day history of right-sided neck and pharyngeal pain that fluctuated in severity. Her medical history was notable for bronchial asthma, hypertension, and atopic dermatitis; active medications included a vilanterol trifenatate fluticasone furoate, esomeprazole, dupilumab, suvorexant, and amlodipine. On examination, her heart rate was 77/minute and blood pressure was 151/71 mm Hg. Laboratory testing results were as follows: white blood cell count, 7.0 × 10³/μL, C-reactive protein, 1.22 mg/dL; triglyceride, 197 mg/dL; low-density lipoprotein, 179 mg/dL; and haemoglobin A1c, 7.3%. US of the neck showed thickening of the outer wall of the right common carotid artery (CCA) with surrounding hypoechoic tissue; probe tenderness was noted in this area during the examination. There was no clear evidence of vascular dissection. Intravascular blood flow was preserved (Figure 1, Supplementary video—see online supplementary material). The left side of the neck was unremarkable on US. Further investigation using contrasted computed tomography was deferred because of the patient’s asthma. Magnetic resonance angiography (MRA) demonstrated no evidence of stenosis or dissection of the right CCA. No features suggestive of Takayasu arteritis, such as wall thickening or signal change at the base of the aortic arch branches, were noted (Figure 2). Fat suppression T2-weighted imaging using a short tau inversion recovery (STIR) sequence showed high signal intensity surrounding the artery, suggesting perivascular oedema. This was not present on MRI performed 7 months prior for a different purpose. Diffusion-weighted imaging (DWI) showed no areas with diffusion restriction (Figure 3).

**Figure 4.**
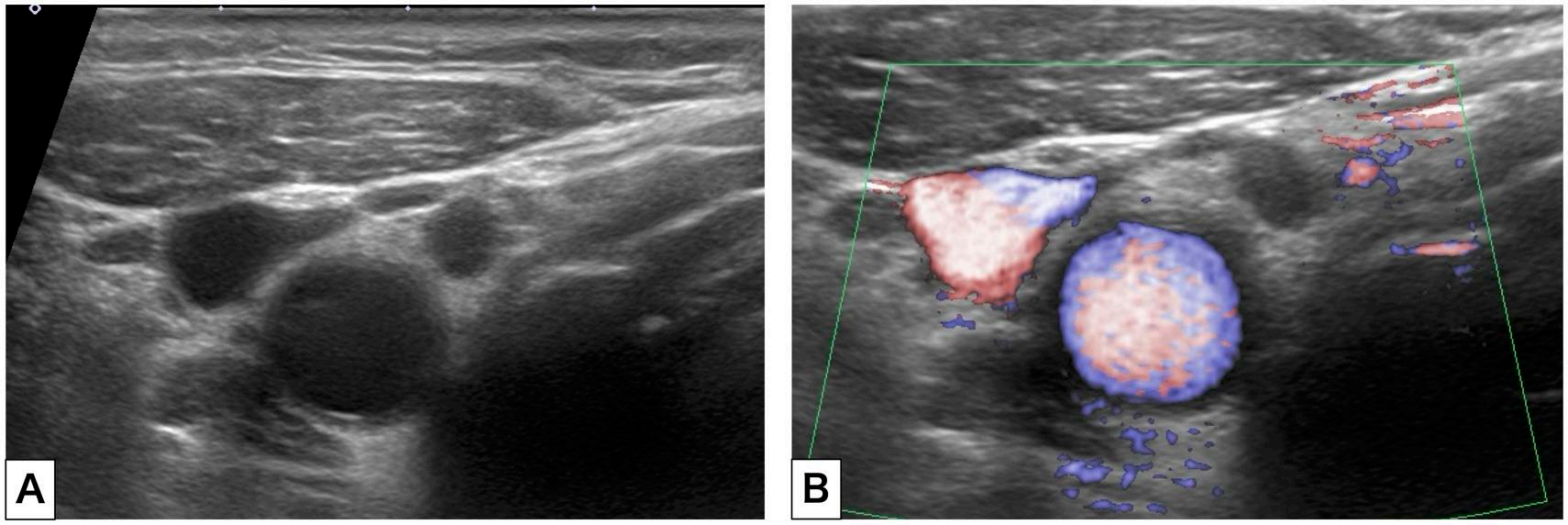
Follow-up B-mode (A) and colour Doppler (B) axial ultrasonography of the neck showed reduction in the thickness of the right common carotid artery and disappearance of the hypoechoic area around the artery.

TIPIC syndrome was diagnosed and the patient was observed in another nearest hospital with a neurosurgery department overnight. She was discharged the following day without complications or new medications. Follow-up US 1 week later showed complete resolution of the previously observed wall thickening and surrounding changes (Figure 4). Her symptoms had also resolved, supporting the diagnosis of TIPIC syndrome.

## Discussion

TIPIC syndrome is a rare and under recognized clinical entity characterized by acute unilateral neck pain localized to the carotid artery region. First described by Fay^1^ in 1927 as “atypical neuralgia,” the condition was formally included in the International Classification of Headache Disorders in 1988.^2^ However, because of its ambiguous diagnostic criteria, it was later removed from the classification in 2004.^3^ Recently, advances in imaging have led to the recognition of TIPIC syndrome as a distinct diagnostic entity with consistent radiological features.^4–6^

Clinically, TIPIC syndrome typically presents with acute, throbbing, or pressure-like pain localized over the carotid bifurcation, often associated with focal tenderness. Some patients may experience referred symptoms such as headache, otalgia, or dizziness caused by stimulation of the pericarotid sympathetic plexus.^6^ Because systemic inflammatory markers are generally normal or only mildly elevated, imaging studies are crucial for making the diagnosis.^4–7^ Histopathological evidence is limited, as biopsies are rarely performed; however, lymphocytic infiltration with scattered neutrophils, vascular proliferation, and early fibrosis has been reported, suggesting a chronic inflammatory process centred in the adventitia.^8^

US typically demonstrates eccentric mural thickening or hypoechoic perivascular changes at the site of maximal tenderness, without significant luminal narrowing.^4–7^ However, these subtle findings may be overlooked. In our patient, US demonstrated perivascular abnormalities which were further investigated using MRI to establish the correct diagnosis. MRI is particularly valuable because it delineates perivascular soft tissue thickening with high signal intensity on fat-suppressed T2-weighted and contrast-enhanced T1-weighted imaging.^4–7^^,^^9^ Reports of low signal intensity on non-fat-suppressed T2-weighted imaging highlight the importance of fat suppression for accurate assessment, given the abundance of surrounding fatty tissue.^10^ Diffusion-weighted imaging has rarely been reported in TIPIC syndrome; in our patient, the absence of restricted diffusion despite high T2 signal suggested oedema rather than cellular proliferation. These findings support the interpretation of a benign, inflammatory, and self-limiting process. Importantly, our patient could not undergo contrast-enhanced computed tomography because of asthma, and the diagnosis was established with unenhanced MRI, underscoring the clinical value of this approach in patients in whom contrast is contraindicated. Although perivascular lesions may also enhance after contrast administration, the presence or absence of this finding does not change the diagnosis.

According to Lecler et al.’s diagnostic criteria for TIPIC syndrome,^4^ in addition to the eccentric perivascular lesions seen in this case, lesions must be excluded by imaging (Table 1). The differential diagnosis of unilateral carotid pain includes arterial dissection, large-vessel vasculitis such as Takayasu arteritis, atherosclerotic plaque, fibromuscular dysplasia, and thrombosis.^7^^,^^11^ Given that each differential diagnosis may present with similar arterial wall thickening on imaging, careful and detailed imaging evaluation is required. Unlike TIPIC syndrome, these conditions generally produce luminal narrowing or hemodynamic changes, which can be excluded by Doppler US and magnetic resonance angiography (Table 2). Thus, TIPIC syndrome remains largely a diagnosis of exclusion, requiring careful clinical and radiological correlation.

**Table 1.** Criteria for TIPIC syndrome by Lecler et al.^4^

(1) Presence of acute pain overlying the carotid artery, which may or may not radiate to the head.
(2) Eccentric perivascular infiltration on imaging.
(3) Exclusion of another vascular or nonvascular diagnosis with imaging.
(4) Improvement within 14 days either spontaneously or with anti-inflammatory treatment.

**Table 2.** Imaging findings for differential diagnosis of TIPIC syndrome.

Differential diagnosis	Imaging findings not seen in TIPIC syndrome
Arterial dissection	Flap in artery. If the false lumen is occluded, artery luminal narrowing.
Large-vessel vasculitis	Artery luminal narrowing. Wall thickening at the aortic arch branches.
Fibromuscular dysplasia	Artery luminal narrowing and irregularities.
Thrombosis	Artery luminal narrowing.

Therapeutic strategies are conservative. Nonsteroidal anti-inflammatory drugs are usually effective and lead to rapid improvement; corticosteroids are reserved for refractory cases.^6^ In our patient, symptoms resolved spontaneously without pharmacologic treatment, and follow-up US confirmed complete regression of perivascular inflammation. Although most patients experience a self-limiting course within 2-4 weeks, recurrent episodes have been described, highlighting the need for continued clinical awareness and follow-up in selected cases.^12^^,^^13^

In conclusion, TIPIC syndrome is a rare but important cause of unilateral neck pain. Characteristic imaging findings, combined with careful exclusion of alternative vascular disorders, enable an accurate diagnosis, which can avoid unnecessary interventions, as management is generally conservative. Unenhanced imaging can make the diagnosis in patients who cannot undergo contrast-enhanced studies. Appropriate follow-up is important given the potential for recurrence, which is uncommon.

## Learning points

- TIPIC syndrome should be considered in patients with acute unilateral neck pain and focal tenderness over the carotid artery, particularly when other vascular disorders such as dissection or vasculitis have been excluded.
- Characteristic imaging findings include eccentric wall thickening and perivascular soft tissue changes on ultrasonography, and perivascular high signal intensity on fat-suppressed T2-weighted MRI without evidence of luminal narrowing or dissection.
- TIPIC syndrome is a benign and usually self-limiting condition that often resolves with conservative management; recognition of this entity can prevent unnecessary invasive procedures or treatments.

## Supplementary Material

uaag002_Supplementary_Data
